# Auto‐Trending daily quality assurance program for a pencil beam scanning proton system aligned with TG 224

**DOI:** 10.1002/acm2.13117

**Published:** 2020-12-18

**Authors:** Chengyu Shi, Qing Chen, Francis Yu, Jingqiao Zhang, Minglei Kang, Shikui Tang, Chang Chang, Haibo Lin

**Affiliations:** ^1^ New York Proton Center New York NY USA; ^2^ Texas Proton Therapy Center Dallas TX USA; ^3^ California Protons Cancer Therapy Center San Diego CA USA; ^4^ Department of Radiation Medicine and Applied Sciences University of California San Diego

**Keywords:** automation, DQA, PBS, ProBeam, Proton, TG 224

## Abstract

The Daily Quality Assurance (DQA) for a proton modality is not standardized. The modern pencil beam scanning proton system is becoming a trend and an increasing number of proton centers with PBS are either under construction or in planning. The American Association of Physicists in Medicine has a Task Group 224 report published in 2019 for proton modality routine QA. Therefore, there is a clinical need to explore a DQA procedure to meet the TG 224 guideline. The MatriXX PT and a customized phantom were used for the dosimetry constancy checking. An OBI box was used for imaging QA. The MyQA(TM) software was used for logging the dosimetry results. An in‐house developed application was applied to log and auto analyze the DQA results. Another in‐house developed program "DailyQATrend" was used to create DQA databases for further analysis. All the functional and easy determined tasks passed. For dosimetry constancy checking, the outputs for four gantry rooms were within ±3% with room to room baseline differences within ±1%. The energy checking was within ±1%. The spot location checking from the baseline was within 0.63 mm and the spot size checking from the baseline was within −1.41 ± 1.27 mm (left–right) and −0.24 ± 1.27 mm (in–out) by averaging all the energies. We have found that there was also a trend for the beam energies of two treatment rooms slowly going down (0.76% per month and 0.48 per month) after analyzing the whole data trend with linear regression. A DQA program for a PBS proton system has been developed and fully implemented into the clinic. The DQA program meets the TG 224 guideline and has web‐based logging and auto treading functions. The clinical data show the DQA program is efficient and has the potential to identify the PBS proton system potential issue.

## 1 INTRODUCTION

Daily Quality Assurance (DQA) of a machine is an essential and important first step for radiation therapy daily treatment. For proton therapy, since the machine types are different, the DQA procedures are not standardized as photon machines. Limited literature has been published^1^, ^2^, ^3^, ^4^ in previous years about proton machine DQA, however, there are lots of variety for testing tasks, equipment, software, and time spent on the DQA. According to the Particle Therapy Co‐Operative Group (PTCOG, https://www.ptcog.ch/) statistics, in the United States, proton center numbers are increasing in recent years. There are 37 centers (85 gantries) in operation, 6 centers (10 gantries) are under construction, and 6 centers (possibly 11 gantries) are in the planning stage up to July 2020. There are great needs to revisit how to do the DQA for different types of proton modality. On the other hand, previously published task group reports (such as TG 40,^5^ TG 142,^6^ TG 179^7^) by the American Association of Physicists in Medicine (AAPM) are more focusing on photon/electron modalities. A recent published TG report 224^8^ in 2019 has proposed tasks for DQA of proton modalities subject to adoption for a center to fit the need of the centers’ machine type. Proton therapy technique has further developed in recent years. The pencil beam scanning (PBS) technique has become a future trend for intensity‐modulated proton therapy (IMPT). Therefore, the TG 224^8^ report guideline needs to be adopted for the unique features of a PBS proton system.

Daily quality assurance tasks are usually less than monthly and annual QA tasks, however, the DQA data are bigger than monthly and annual QA with time accumulated. There is also a need to automatically collect the “big” data and analyze the trend of the “big” data. It will be also good to predict machine performance ahead of time for maintenance to avoid longer machine downtime. There is a clinical need for developing software to automatically run in the background to put the DQA data into a database and show each parameter trend with time. Few DQA literature combined the DQA data with automation and Artificial Intelligent (AI) implementation,^9^ although there have been reports on machine learning applications in proton dose verifications,^10^, ^11^, ^12^ Linac QA,^13^, ^14^, ^15^ patient‐specific QA in radiotherapy.^16^, ^17^, ^18^, ^19^

Based on the clinical urgent need for DQA procedures, automation, and AI implementation, an auto trending DQA program alignment with TG 224 guideline has been developed at New York Proton Center (NYPC). The program has been run for a year for a PBS proton system. In the following sections, we will report the DQA program and the outcome of the DQA program in detail.

## 2 MATERIALS AND METHODS

### 2.1 DQA tasks and general workflow

The following tasks were tested for DQA: safety interlocks, kV X‐ray/CBCT image‐guided radiation therapy (IGRT) system tests, and proton beam quality consistency. The tasks, personnel to perform the tasks and checking, and the tolerances are listed in Table 1.

**Table 1:** DQA tasks, personnel responsibility, and tolerances.

Parameters	Performed by	Supervised by	Acceptability	TG 224 compatible
Safety checks and interlock checks				
Audio–visual monitor	Therapist	QMP*	Functional	√
Radiation monitor	Therapist	QMP	Functional	√
Collision laser guard on the gantry head	Therapist	QMP	Functional	√
Collision laser guard in the snout head	Therapist	QMP	Functional	√
Collision touch guard on the snout cover	Therapist	QMP	Functional	√
Collision touch guard on the couch arms	Therapist	QMP	Functional	√
Radiation beam on indicator	Therapist	QMP	Functional	√
kV X‐ray beam indicator	Therapist	QMP	Functional	√
Search/clear button	Therapist	QMP	Functional	√
Door interlock	Therapist	QMP	Functional	√
Proton beam on indicator	Therapist	QMP	Functional	√
Pause beam button	Therapist	QMP	Functional	√
IGRT system checks				
kV/kV 2D/3D match	Therapist	QMP	±2 mm	√
CBCT 3D/3D match	Therapist	QMP	±2 mm	√
Lasers position accuracy	Therapist	QMP	±2 mm	√
Proton beam quality consistency checks: compare with the commissioning baseline				
Range	Therapist	QMP	1 mm	√
Spot position	Therapist	QMP	±1.5 mm	√
Spot size	Therapist	QMP	±10%**	***
Output	Therapist	QMP	±3%	√
Field symmetry	Therapist	QMP	±2 %	√****
Field flatness	Therapist	QMP	±2 %	√****
Field size	Therapist	QMP	±2 mm	√****
IC2 (2nd MU) counts	Therapist	QMP	±2%	√

*QMP refers to Qualified Medical Physicist.

**10% is less than 1 mm.

***TG 224 has no requirement for spot size DQA.

****Optional for pencil beam scanning proton system.

**Fig. 1:**
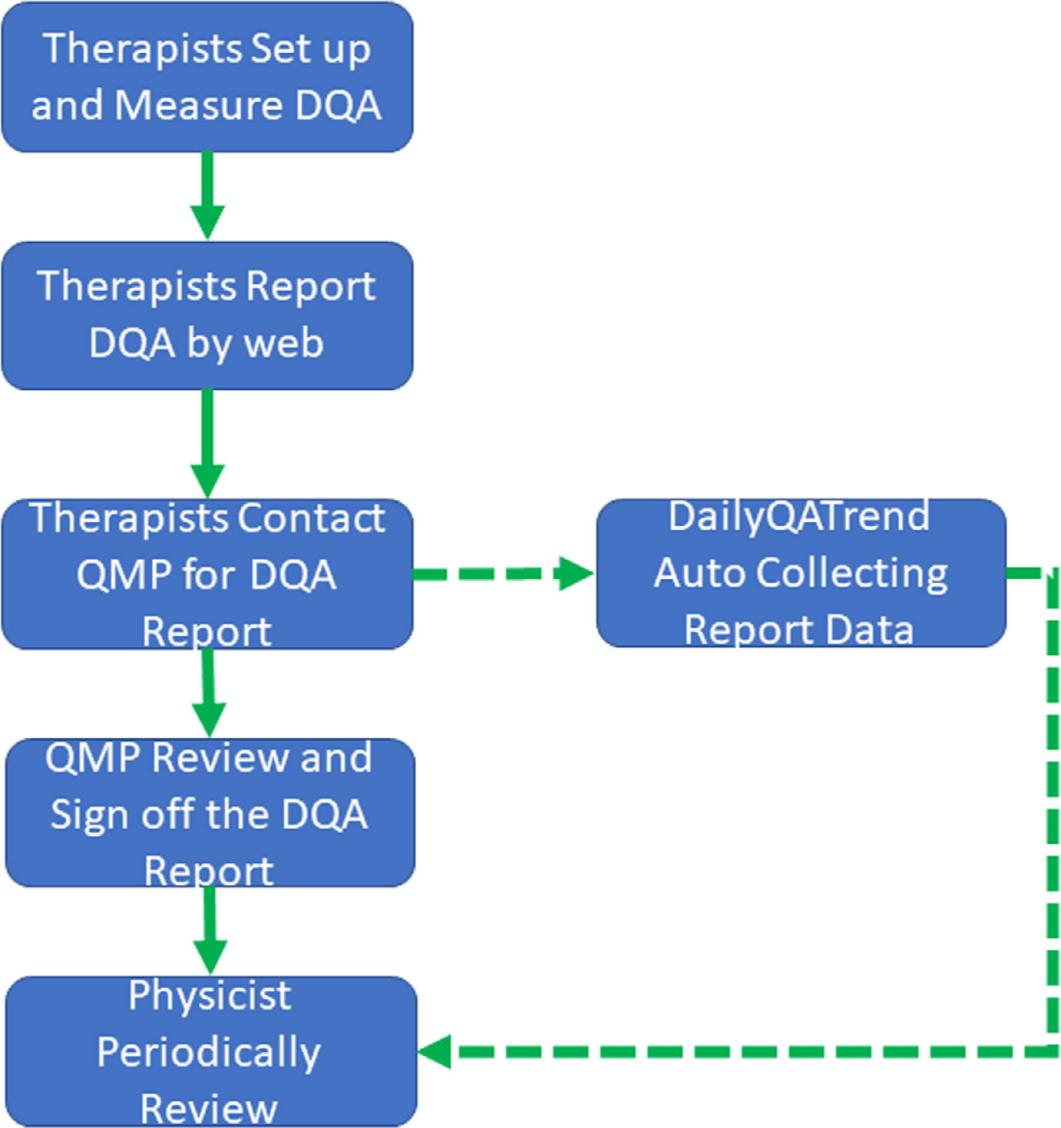
General DQA workflow at the New York Proton Center.

Comparing with the TG 224^8^ DQA procedure for proton therapy, the tasks are suitable for a PBS proton system and alignment well with the TG 224 guideline. Fig. 1 shows the general DQA workflow at NYPC.

### 2.2 Hardware

**Fig. 2:**
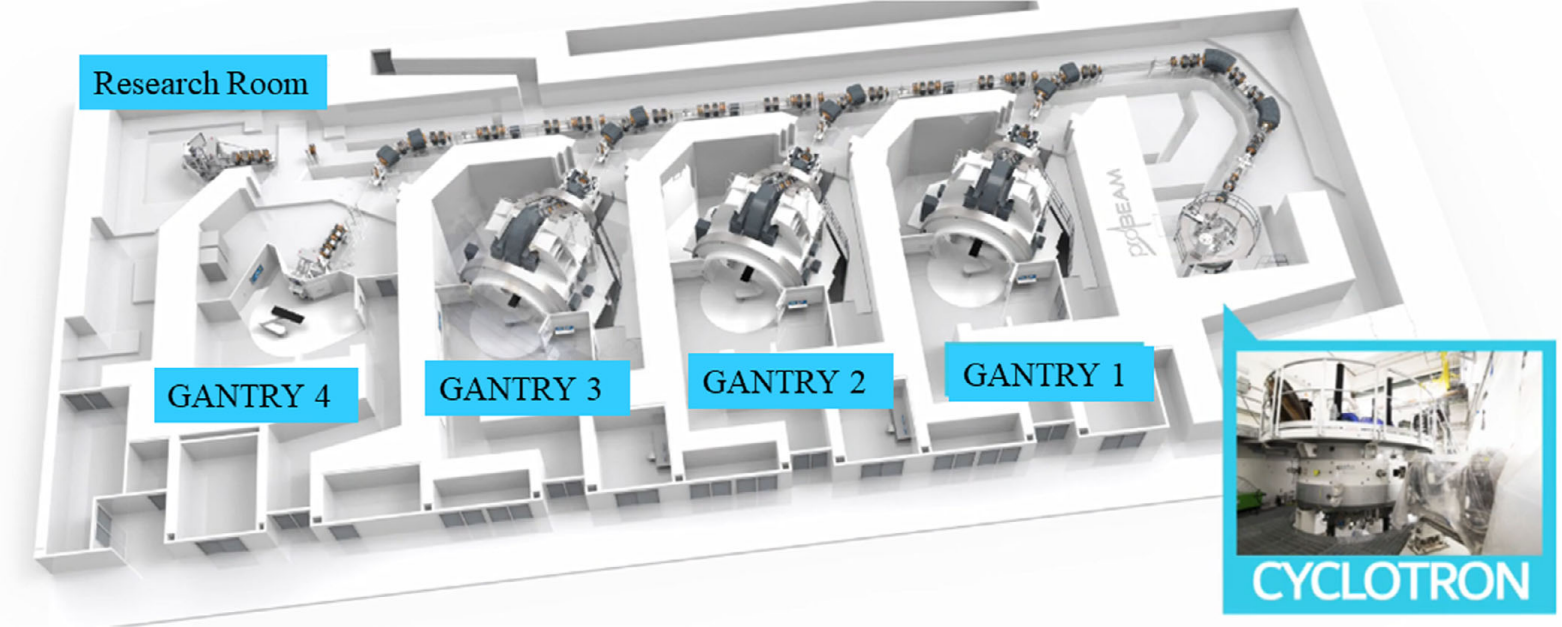
New York Proton Center treatment room distribution: three rotation gantry rooms (Gantry 1–3), one fixed beam room (Gantry 4), and one research beam room.

New York Proton Center has a PBS proton system, Probeam^TM^, manufactured by the Varian Medical System (Palo Alto, CA, USA). The system has three rotation gantry rooms, one fix beam room, and one research beam room as illustrated in Fig. 2. The center was planned 10 years ago and was open in Aug 2019.

For DQA, MatriXX PT manufactured by IBA dosimetry (Schwarzenbruck, Germany) was used for dosimetry measurement. The MatriXX PT is an ion chamber‐based 2D array device with a pixel spacing of 7.6 mm, ion chamber volume of 32 mm,^3^ and an electrode gap of 2 mm. The array comes with factory uniformity calibration. However, it must go through an absolute dose calibration to provide accurate dose measurements. Once the proton machine was calibrated according to the IAEA TRS 398^20^ protocol using an Accredited Dosimetry Calibration Laboratory (ADCL) calibrated ion chamber, the MatriXX PT were placed in the same reference conditions (temperature, pressure, depths) and the same proton beam (used for TRS 398, e.g., 180 MeV at 2 cm depth) was used to irradiate the MatriXX PT. The dose reading from ADCL calibrated ion chamber was used to cross calibrate the MatriXX PT. The calibration procedure was repeated during the annual QA of each treatment room immediately after the TRS 398^20^ protocol done, after a major repair, or as frequently as needed.

**Fig. 3:**
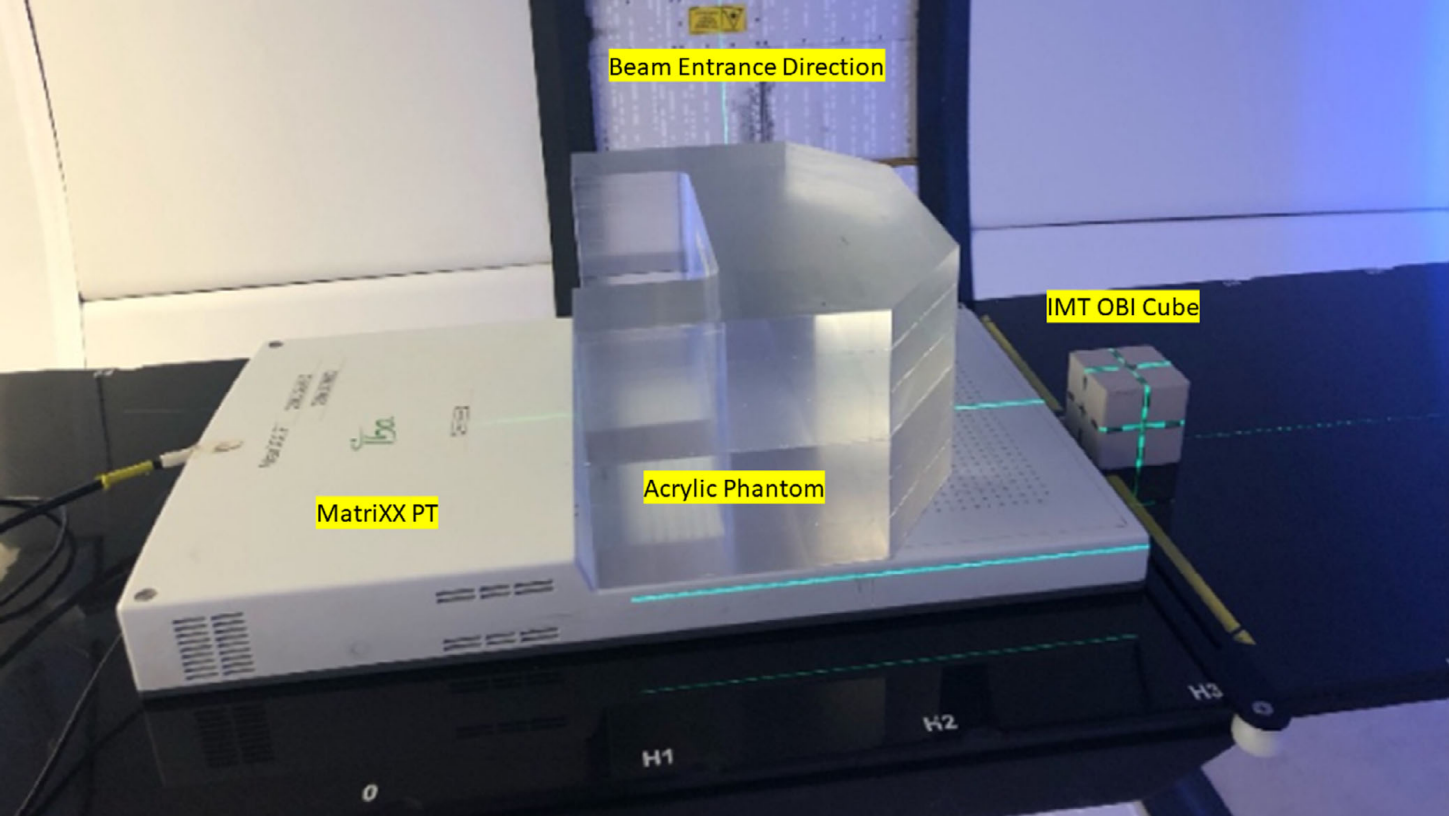
The hardware placement layout of DQA.

An in‐house developed acrylic phantom was used with MatriXX PT together. The acrylic phantom was used as a buildup for Spread Out Brag Peak (SOBP). The phantom is 15 cm thick with 32 cm wide and 22 cm long. The wide dimension matched with MatriXX PT well. The phantom shape was customized to allow the DQA dosimetry plan pattern measurable for different proton energies. An OBI cube from Integrated Medical Technologies (IMT Inc, Troy, NY, USA) was used for imaging QA purposes. The typical setup image for DQA is illustrated in Fig. 3.

### 2.3 Dosimetry pattern design

**Fig. 4:**
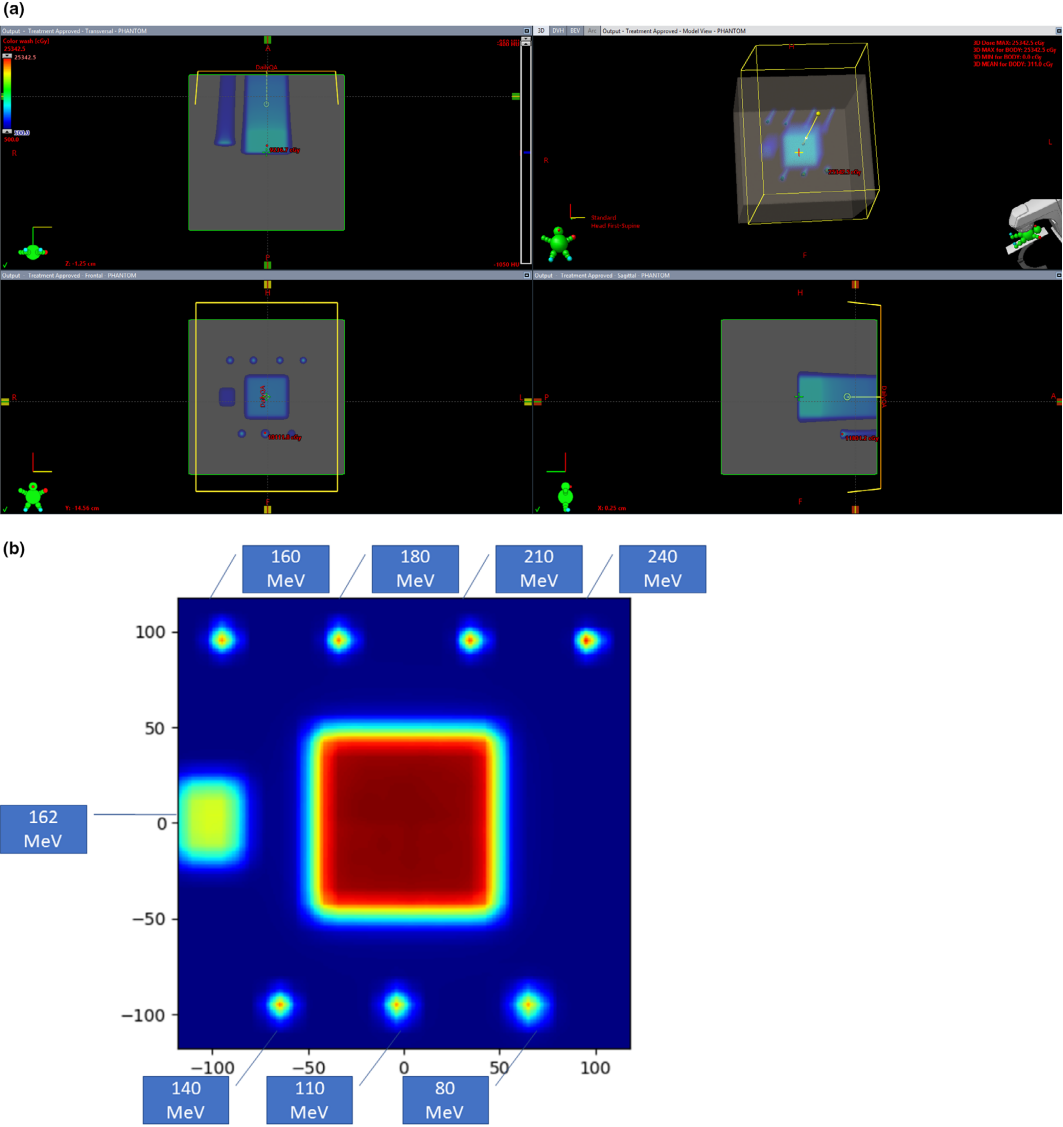
Dosimetry test pattern for DQA (a) and expected result from MatriXX PT (b).

A test plan pattern was designed for MatriXX PT as shown in Fig. 4(a). The test pattern can be used to test range consistency, spot size consistency, spot position consistency, output, field flatness consistency, field symmetry consistency, and field size consistency. Pristine beam energy 80, 110, 140, 160, 180, 210, and 240 MeV proton spots were designed to shoot around two square areas. The smaller square area (3 × 4 cm^2^ at 50% isodose line) was for single energy 162 MeV proton beam range test and the larger square area (10 × 10 cm^2^ at 50% isodose line) was intensity modulated for energy ranges of 145–173 MeV and was used for output test. The expected MatriXX PT measured image is shown in Fig. 4(b).

### 2.4 Software system

**Fig. 5:**
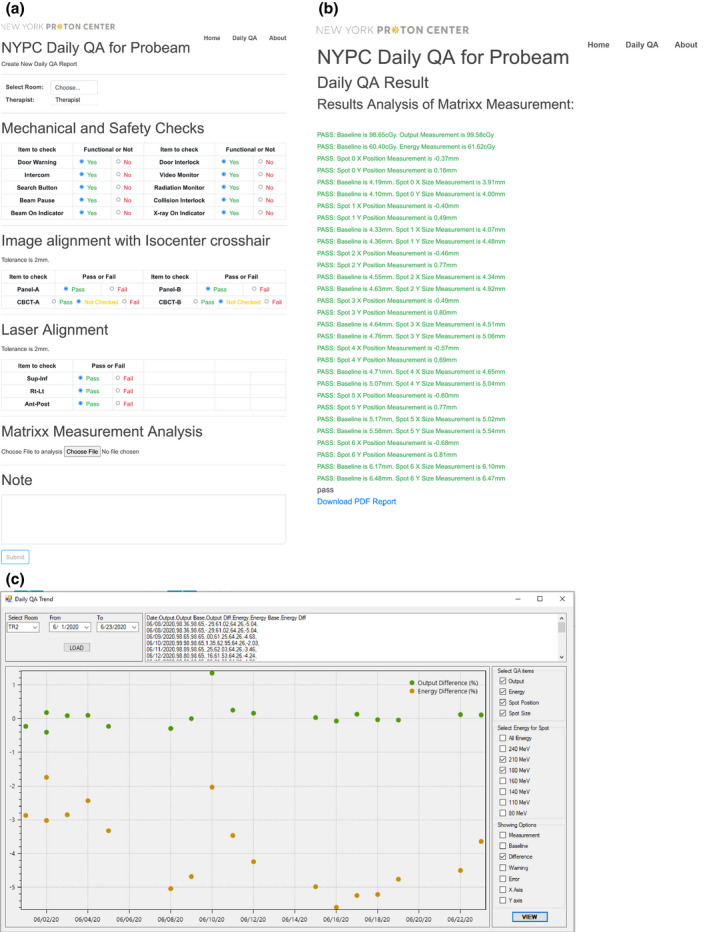
Software system for DQA analysis. (a) web‐based python application for DQA logging; (b) application report; (c) interface of the “DailyQATrend” software.

MyQA software (IAB dosimetry, Schwarzenbruck, Germany) was purchased together to collect data from MatriXX PT hardware. A python‐web application was developed to log the DQA result and provide feedback to the therapists. The report can be in PDF format and will be saved to a folder in a shared drive. An in‐house software called “DailyQATrend” was developed to auto collect daily QA PDF report and put the extracted data into a database to show the QA trend. The main reasons for developing our software vs implementing commercial software are considering the time efficiency, integration of the system, and background calculation functions. Fig. 5 shows the python‐web application interface, the report from the web application, and the interface of “DailyQATrend” software. The python‐web application will do proton beam consistency analysis on the background. The program can analyze the data based on the exported measurement file from the MatriXX PT. The following parameters were calculated by the application.

Range consistency is to ensure the consistency of the proton range (energy) for a designated proton beam. The proton range is defined at the position where the dose has decreased to 80% of its maximum dose in the distal dose falloff, which physically corresponds to a depth that 50% of mono‐energetic proton stops. This test delivers a designed proton beam distal Brag Peak with a range of 15 cm in the acrylic phantom and measures its dose at the depths of 15 cm. The acrylic phantom is 15 cm thick (water equivalent thickness (WET) is 17 cm) and range variation will cause the measured dose larger difference (such as 1 mm will have ~12% dose difference) from the baseline value acquired during the commission.

Machine output is determined by the center pattern in Fig. 4b. MatriXX PT was cross‐calibrated with an ADCL calibrated ion chamber and the pattern reading exported was absolute dose measured by MatriXX PT. A square 3 × 3 cm^2^ in the center was averaged by the python‐web application and reported as absolute dose reading. The output was compared with the baseline value during the commission.

Spot location and size for each pristine proton energy was fitting by a Gaussian function:

(1) $$f\left(x,y\right)=Ae^{\left(‐\frac{1}{2}\left({\left(\frac{x‐\mu_{x}}{x}\right)}^{2}+{\left(\frac{y‐\mu_{y}}{y}\right)}^{2}\right)\right)}$$

Here the coefficient A is the amplitude. The fitting parameters of expected value µ_x_, µ_y_, and variance σ_x_, σ_y_ were spot location and size in x (left–right) and y (in–out) direction respectively. The fitting is based on the exported MatriXX PT file with 1 mm resolution data. The calculated values will be compared with the baseline values for the spot.

According to ICRU No. 78,^21^ the lateral flatness (in percent) is defined as:

(2) $$LateralFlatness\text{\textbackslash{}\%}\;=\frac{\left(d_{\max}‐d_{\min}\right)}{\left(d_{\max}+d_{\min}\right)}\times 100$$

where d_max_ and d_min_ are the maximum and minimum absorbed dose values in the beam profile measured around the central axis in the IMPT large square area in Fig. 4(b).

The lateral symmetry (in percent) is defined as:

(3) $$LateralSymmetry\%=\frac{\left(D1‐D2\right)}{\left(D1+D2\right)}\times 100$$

where D1 and D2 are the sampled absorbed doses in the beam profile measured around the central axis in the IMPT large square area in Fig. 4(b). The field size was measured for the IMPT large square area in Fig. 4(b) based on the 50% isodose line.

## 3 RESULTS

Some tests (such as functional tests) in Table 1 will have instant results and can be determined by a therapist, therefore, the python‐web application will provide a chance for a therapist to put down Pass/Fail information. The dosimetry results are hard to judge, and some cannot be determined by just knowing the number, then it will need further assistant from the python‐web application or even the “DailyQATrend” program. The results about output, energy, spot position, and spot size are shown in the following.

**Fig. 6:**
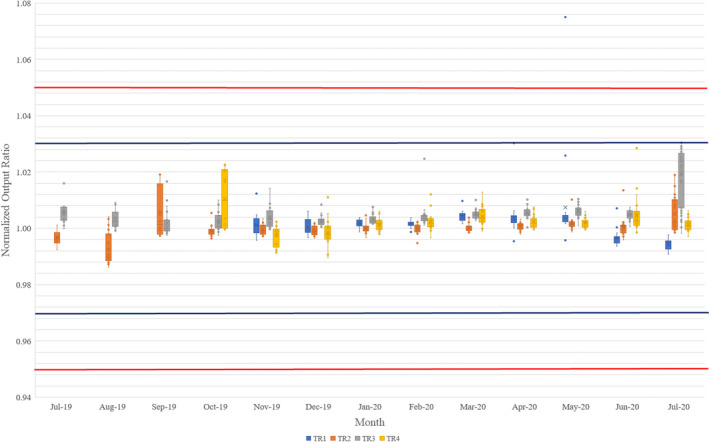
Output changes for four gantry rooms. The box‐and‐whisker plot (https://en.wikipedia.org/wiki/Box_plot) shows 75% of the data range, median range, and 25% of the data range. The top bar shows the 1.5 box size above the 75% of the data range, and the bottom bar shows the 1.5 box size below 25% of the data range. Outside of the top and bottom bars are outliers. The blue line represents 3% as the warning zone and the red line represents 5% as the failure zone.

The daily output values were shown over the month in Fig. 6 with the box‐and‐whisker plot. The treatment rooms #2 and #3 (TR2 and TR3) were commissioned for clinical usage earlier followed by treatment rooms #4 and #1 (TR4 and TR1). The outputs overall are within ±3% range from baseline with certain fluctuations. The four rooms’ baseline differences are within ±1% so that we can transfer patients if other hardware allows, such as TR4 has fixed gantry so that it cannot deliver a plan with multi gantry angles. Figure 6 data were averaged by a month, 1 day output jump (such as ±2%–3%) will cause larger uncertainties for the data as shown as the May 2020 data of TR4. TR3 output was adjusted in July 2020 by considering the annual QA result.

**Fig. 7:**
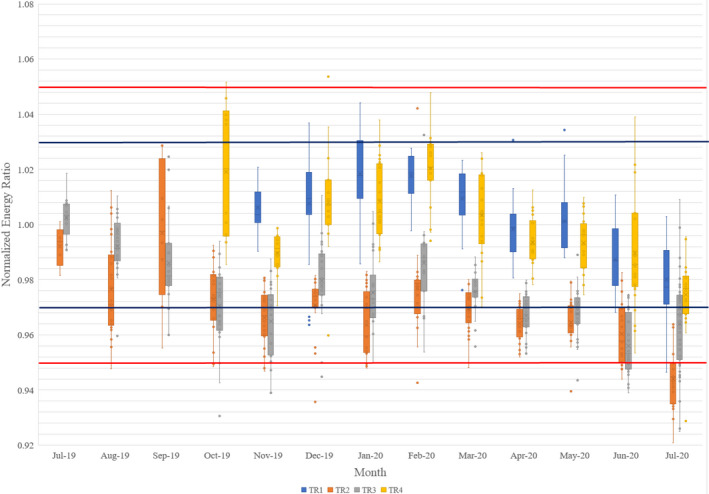
Energy changes for four gantry rooms. The box‐and‐whisker plot (https://en.wikipedia.org/wiki/Box_plot) shows 75% of the data range, median range, and 25% of the data range. The top bar shows the 1.5 box size above the 75% of the data range, and the bottom bar shows the 1.5 box size below 25% of the data range. Outside of the top and bottom bars are outliers. The blue line represents 3% as the warning zone and the red line represents 5% as the failure zone.

The daily energy check values were shown over the month in Fig. 7 with the box‐and‐whisker plot. The energy is very within the tolerance overall months. The TR4 room has larger uncertainties during the first commissioning period and changed certain hardware during that period. The energy of TR4 became stable after a couple of months. It is also interesting to notice that TR2 and TR3 energy check have the trend to go down slowly. The energy check went down 0.76% per month and 0.48% per month for TR2 and TR3, respectively, by fitting the data with linear regression. The real reason is still unknown, and we keep watching the data closely and work with the vendor to find out the real reason. The other two rooms have not shown a similar trend yet.

**Fig. 8:**
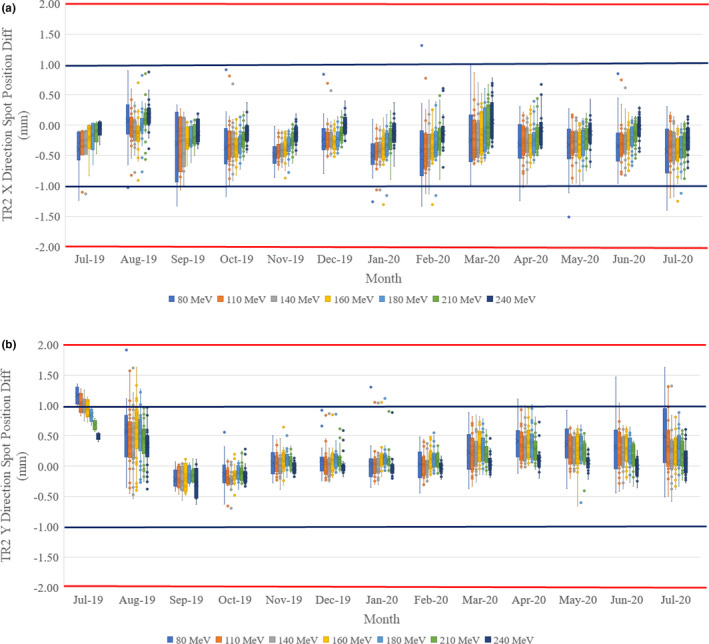
Room TR2 spot location changes averaged by month: (a) X (left–right) direction; (b) Y (in–out) direction. The box‐and‐whisker plot (https://en.wikipedia.org/wiki/Box_plot) shows 75% of the data range, median range, and 25% of the data range. The top bar shows the 1.5 box size above the 75% of the data range, and the bottom bar shows the 1.5 box size below 25% of the data range. Outside of the top and bottom bars are outliers. The blue line represents 1 mm as the warning zone and the red line represents 2 mm as the failure zone.

A typical spot position difference plot is shown in Fig. 8(a) and Fig. 8(b) with the box‐and‐whisker plot. Here the TR2 room spot position differences were shown for all sampled energies for x‐direction (left–right) and y‐direction (in–out). The spot position differences are overall less than 0.63 mm by average. The higher energy will have less spot position differences and smaller uncertainties. The possible reason is that the higher energy will be less scattered by the air and detector itself. Another potential reason may be the lower energy spot is more sensitive to the current variations of the magnetic field. There is also a trend for x‐direction the position will be negative and for y‐direction the position will be positive. It may be due to the therapists’ habit to place and align the detector and phantom with a symmetric error. It is possible that the spots are not symmetric as a real Gaussian fitting and the fitting parameter will tend to lean in one direction.

**Fig. 9:**
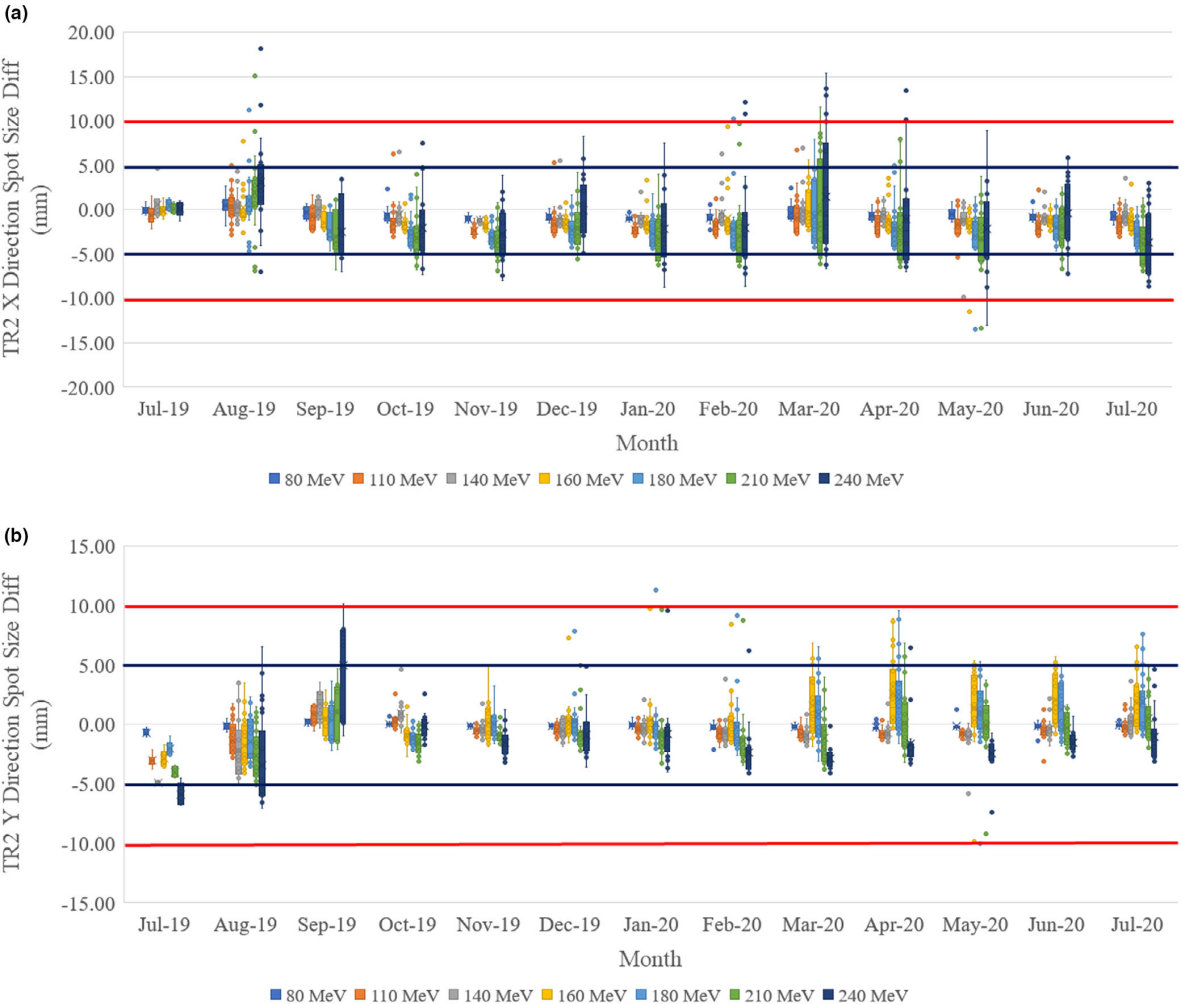
Room TR2 spot size changes averaged by month: (a) X (left–right) direction; (b) Y (in–out) direction. The box‐and‐whisker plot (https://en.wikipedia.org/wiki/Box_plot) shows 75% of the data range, median range, and 25% of the data range. The top bar shows the 1.5 box size above the 75% of the data range, and the bottom bar shows the 1.5 box size below 25% of the data range. Outside of the top and bottom bars are outliers. The spot size is depending on the energy and 10% is used as an acceptable tolerance. The blue line represents 5 mm and the red line represents 10 mm for reference purpose.

A typical spot size difference plot is shown in Figs. 9(a) and 9(b) with the box‐and‐whisker plot. Here the TR2 room spot size differences were shown for all sampled energies for x‐direction (left–right) and y‐direction (in–out). The spot size was determined by fitting the spot shape with a Gaussian function. The σ in Eq. (1) is used as spot size. For the x‐direction, it shows a smaller spot size (−1.41 ± 1.27 mm by average all energies) than the baseline. It also shows the higher energies have larger differences from the baseline and larger uncertainties in the x‐direction. For the y‐direction, the spot size differences are less (−0.24 ± 1.27 mm by average all energies) from the baseline. The higher energies also show larger uncertainties. The higher energy will have sharper and smaller spot size, which is more sensitive to the fitting and cause larger uncertainties in both x‐ and y‐direction. By observing Fig. 4b in detail, the x‐direction spot profiles have wider and unsymmetrical distribution, especially for the higher energies (such as 160, 180, 210, 240 MeV), which will cause the Gaussian fitting to have larger uncertainties.

## 4 DISCUSSION

The whole daily DQA system including the hardware and software has been successfully running since the center was open in Aug 2019. The system is also transferable to another similar hardware setting with some tune for baseline information. The whole program is well aligned with the TG 224 requirement and some tasks are even additional and suitable for a PBS proton system. The whole DQA will take about 30 min for a therapist to finish all tasks and notify an onsite physicist for review and approval. It is a fully implemented and efficient program universally adoptable.

The customized U shape phantom matching with MatriXX PT well. The current phantom will test only one energy (162 MeV). However, the phantom can be further adjusted to have a step‐wedge shape which will test more energy ranges. The composite pattern derived from the DQA shown in Fig. 4(b) can be treated as an IMPT plan. The pattern can be further analyzed by using tighter (1%/1 mm with 10% threshold and 90% passing rate) gamma index^22^ parameter or Structural SIMilarity (SSIM) index.^23^ It will be easy for a program such as the python‐web application to do so and automatically email the result to the medical physicist group as records. The “DailyQATrend” can be further developed to auto trending the DQA results and provide certain maintenance suggestions.

The DQA results have certain uncertainties. For example, the positioning of the measurement devices is subject to affect position uncertainties. Internal sensitivity tests have been done and we found that the results will show warnings and failures if the setup errors are more than 2 mm in the left–right direction or in–out direction. The Gaussian fitting parameters will affect the spot location/size uncertainties. For the sensitivity of using MatriXX PT to test the beam spot size and spot location, a previous study has shown the feasibility.^24^ The spot location is not sensitive to the MatriXX PT ion chamber spacing. However, the spot size might be sensitive to the ion chamber spacing of MatriXX PT. To achieve a 10% spot size, the noise level is allowed to be 2% for lower energy (such as 80 MeV) and 1% for higher energy (such as 240 MeV) proton beam.

The Gaussian fitting method will tolerate a certain noise level. On the other hand, the DQA is a consistency test, which will compare with the baseline instead of the real spot size, therefore, the current MatriXX PT is still fitting into our DQA requirement. The onsite medical physicist still has the responsibility to make the final decision on the machine status suitable for daily treatment or not. The onsite engineers can adjust accordingly if the system needs to be tuned.

## 5 CONCLUSIONS

A DQA program for a PBS proton system has been developed and fully implemented into the clinic. The DQA program meets the TG 224 guideline and has web‐based logging and auto trending functions. The clinical data show the DQA program is efficient and has the potential to identify the PBS system potential issue. The DQA program is also transferable to a similar setting clinic.

## AUTHOR CONTRIBUTION

Dr. Chengyu Shi is the first author and corresponding author of this research, who contributed to the data collection, analysis, and manuscript preparation. Qing Chen, Dr. Ming Lei Kang, Dr. Shikui Tang, Dr. Chang Chang and Dr. Haibo Lin contributed to the program design and deep discussion about the manuscript. Francis Yu did the “AutoTrending” program design. Dr. Jingqiao Zhang did the sensitivity study of the tests and helped to collect the data.

## CONFLICT OF INTEREST

The authors do not have any conflict of interest to declare.
